# Supplementary material for: Semi-crystalline and amorphous materials via multi-temperature 3D printing from one formulation
Source: Nat Commun. 2025 Oct 15;16:8961. doi: 10.1038/s41467-025-64092-9 (PMC12528444; doi:10.1038/s41467-025-64092-9)
Supplement: Supplementary file 1 — Supplementary Information [file 41467_2025_64092_MOESM1_ESM.pdf]

Supplementary Information

Semi-crystalline and amorphous materials via multi-temperature 3D printing from one formulation

Michael Göschl,[§,1] Dominik Laa,[§,2] Thomas Koch,[2] Evan Constable,[3] Xin Liu,[3] Andrei Pimenov,[3] Jürgen Stampfl,[2] Robert Liska,[1] Katharina Ehrmann[1,*]

§, These authors contributed equally.

1Institute of Applied Synthetic Chemistry, Technische Universität Wien, Getreidemarkt 9/163, 1060 Vienna

2Institute of Materials Science and Technology, Technische Universität Wien, Getreidemarkt 9 BE, 1060 Vienna

3Institute of Solid State Physics, Technische Universität Wien, Wiedner Hauptstraße 8, 1040 Wien

** Email: katharina.ehrmann@tuwien.ac.at*

Table of Contents

Supplementary Figures S2

Supplementary Tables S4

Supplementary Discussion S5

Supplementary Figures

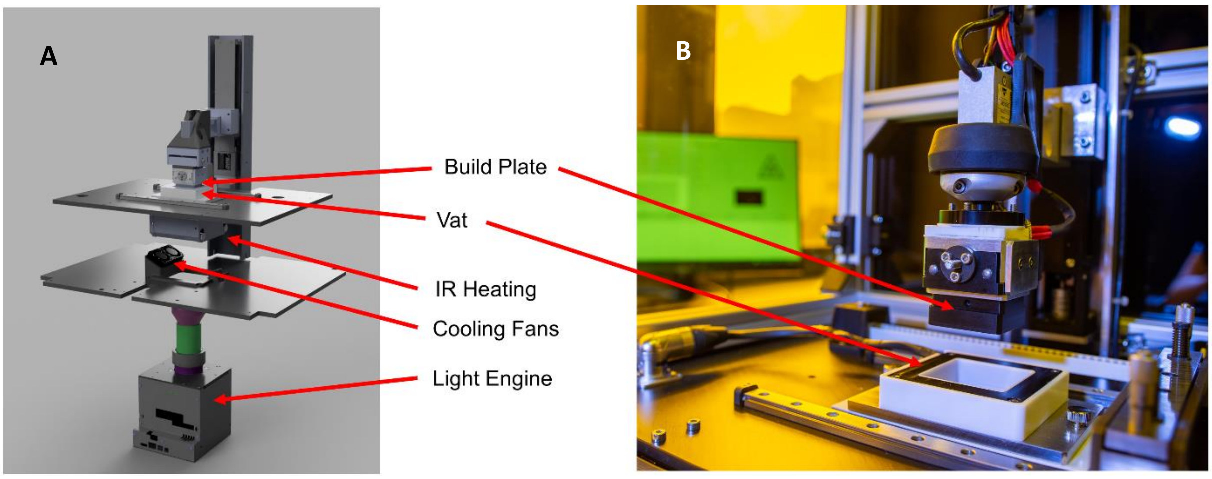

Supplementary Figure 1: *A) Schematic overview of the BP10 printer with equipped cooling system for the vat. B) Photo of BP10.*

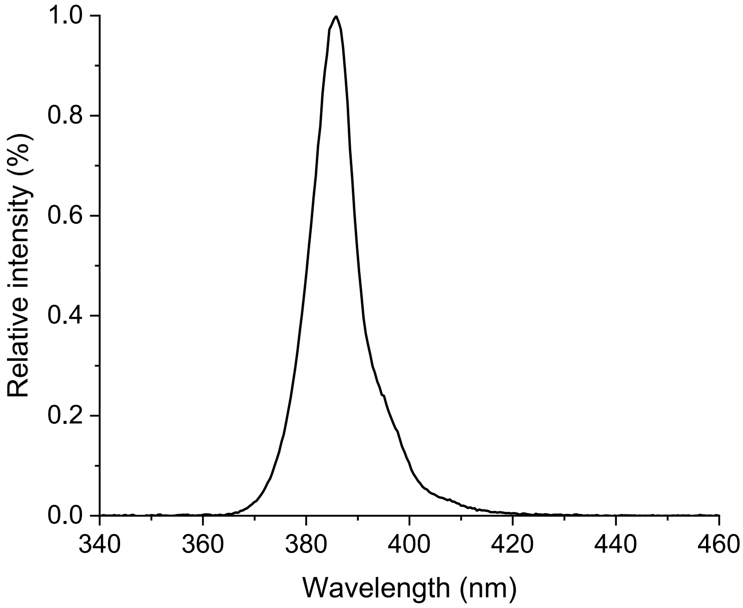

Supplementary Figure 2: *Emission spectrum of the 3D printer BP10 measured in the resin vat.*

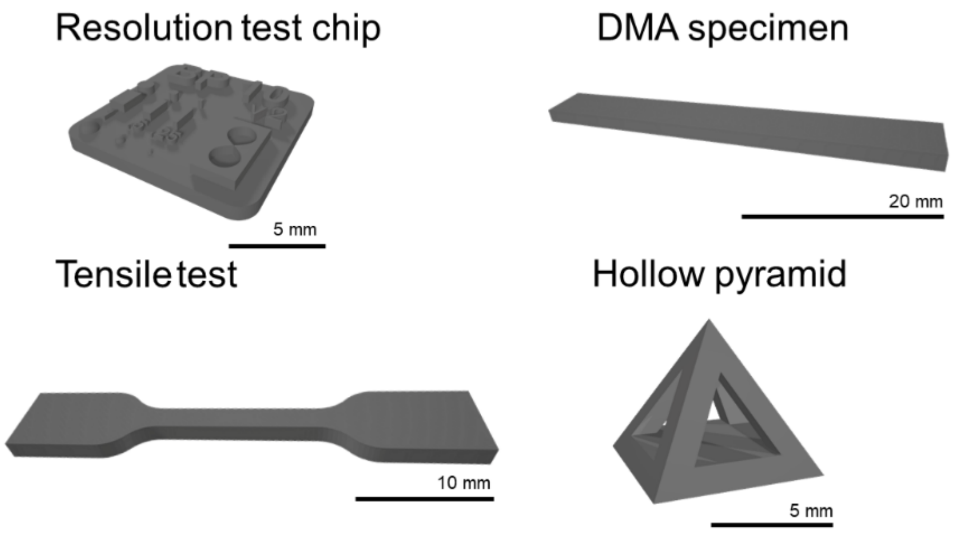

Supplementary Figure 3: *3D models of all 3D printed single-material parts in this work.*

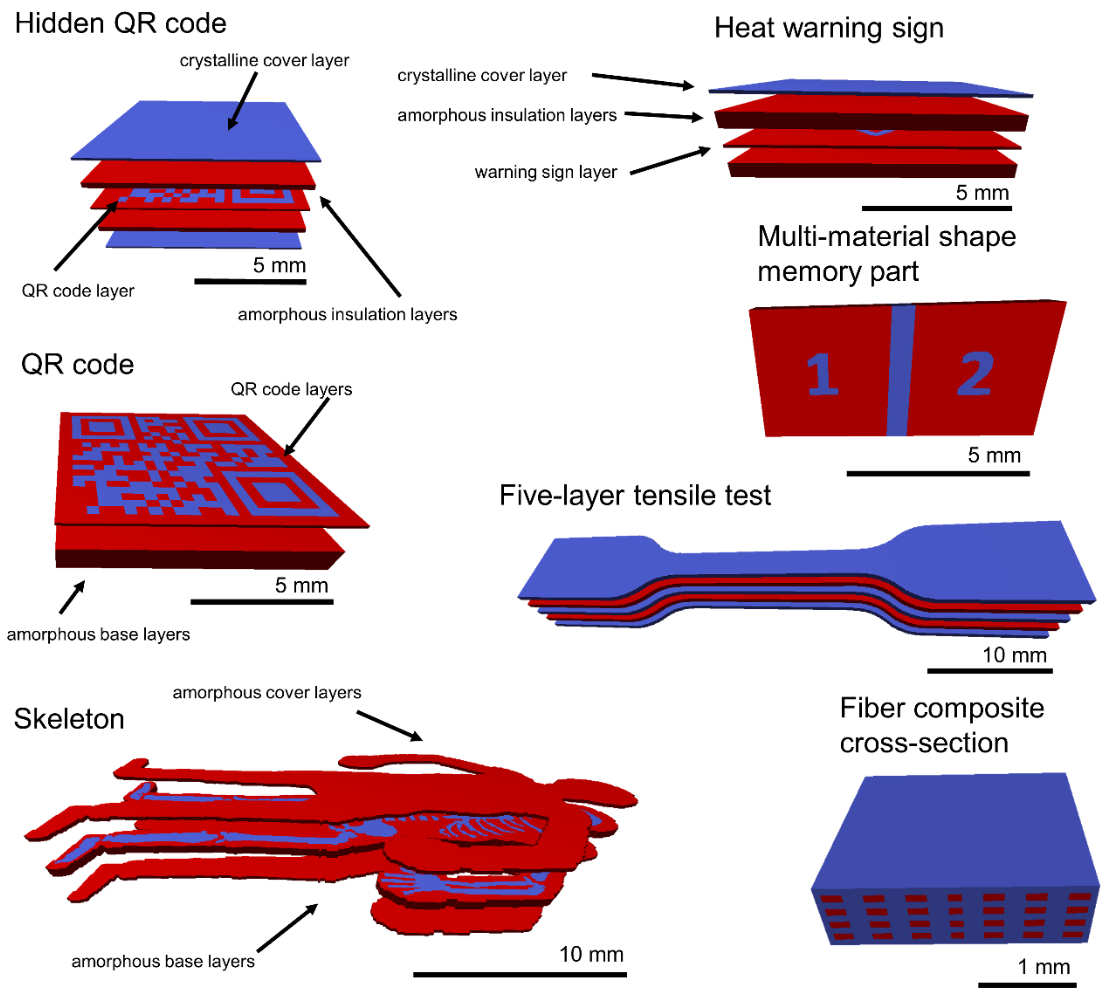

Supplementary Figure 4: *3D models of printed multi-material parts. The sections displayed in red were printed at 100 °C and irradiated for 4.7 s at 80 mW cm^{-2}. The blue sections were printed at 80 °C and irradiated for 24 s at 15.75 mW cm^{-2}.*

Supplementary Tables

Supplementary Table 1: *Formulations used for bulk curing and 3D printing of polymers. Monomers are given in reactive end group percentage (mol% end groups), the photoinitiator diphenyl(2,4,6-trimethylbenzoyl)phosphine oxide (TPO) is given in mole percentage in relation to reactive double bonds, and the stabilizer pyrogallol is given in weight percentage relative to total formulation weight.*

	**BPLC content (mol% end groups)**	**CHTT content (mol% end groups)**	**TPO content (mol%)**	**Pyrogallol content (wt%)**
Bulk curing & 3D printing (except hollow pyramid)	50	50	1	0.2
3D printing of hollow pyramid	50	50	1.5	0.5

Supplementary Table 2: *Polymerization conditions for all specimens bulk cured or 3D printed in this work. Irradiation time for bulk specimens is listed as the total irradiation time per side of the specimen, as it was repeated after the specimens were turned around. Irradiation time for 3D printed specimens is listed as the irradiation time per printed layer. The variations in wavelength were due to equipment availability, as the two-step irradiation procedure for crystalline bulk specimens necessitated the use of two different light sources (which can be used in two different intensity regimes) used in sequence, and the 3D printer was equipped with solely a 385 nm light source of limited irradiation intensity.*

**Sample**	**Temperature (°C)**	**Additional information**	**Wavelength max. (nm)**	**Irradiation time (s)**	**Intensity (mW cm^{-2})**
Bulk crystalline (mild irradiation) 80°C	80	Step 1	405	300	1.5
		Step 2	365	180	290
Bulk crystalline (strong irradiation) 80°C	80	-	365	180	290
Bulk amorphous 110°C	110	-	365	180	290
Temperature gradient print	78-110	Left side	385	4.7	80
		Right side	385	24	15.75
3D printing (crystalline)	80	-	385	24	15.75
3D printing (amorphous 100°C)	100	-	385	4.7	80
3D printing (amorphous 110°C)	110	-	385	4.7	80
3D printing (hollow pyramid)	90	-	385	4.7	80
3D printing (multi-material part without changing temperature)	85	Left side	385	4.7	80
		Right side	385	24	15.75

Supplementary Discussion

Supplementary Note 1: Formulation stability

The temperature stability of the formulation containing 0.2 wt% pyrogallol described in the previous chapter was tested for its temperature stability using melt rheology (**Supplementary Figure 5**). After storage beyond six hours, gelation occurred.

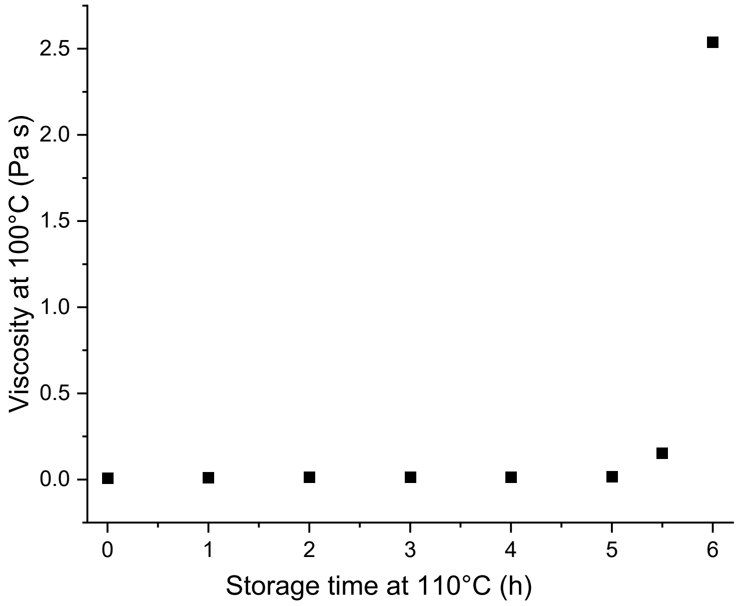

Supplementary Figure 5: *Storage stability of the formulation containing 1 mol% of photoinitiator TPO and 0.2 wt% of stabilizer pyrogallol at 110 °C storage temperature.*

Supplementary Note 2: LC phase analysis of formulation

Polarized optical microscopy was used to analyze the liquid crystalline phase present in the formulations and the temperature gradient 3D print (**Supplementary Figure 6**). Around 79 °C, the formulation melts, forming a liquid crystalline phase, which turns into an isotropic melt at 109 °C. A DSC measurement of the pure monomer BPLC (**Supplementary Figure 7**) reveals phase transitions at relatively similar temperatures to the formulation (77 and 111 °C).

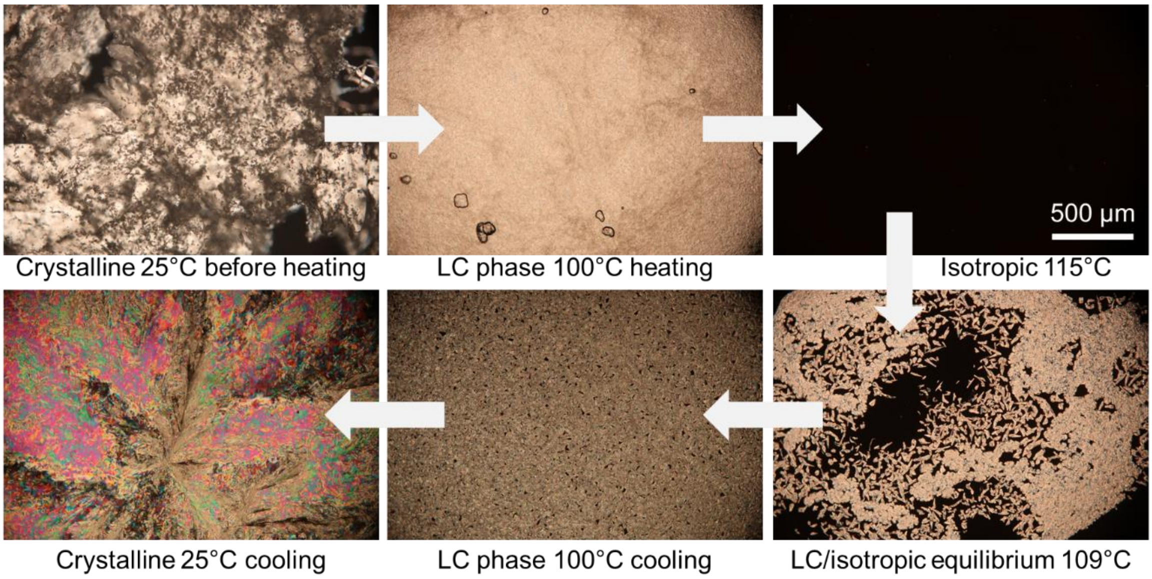

Supplementary Figure 6: *Phase analysis of the formulation consisting of the monomers BPLC and CHTT in a stoichiometrically equal ratio of end groups with 1 mol% of photoinitiator TPO and 0.2 wt% of stabilizer pyrogallol on a polarized optical microscope. A liquid crystalline phase was observed between 79 and 109 °C.*

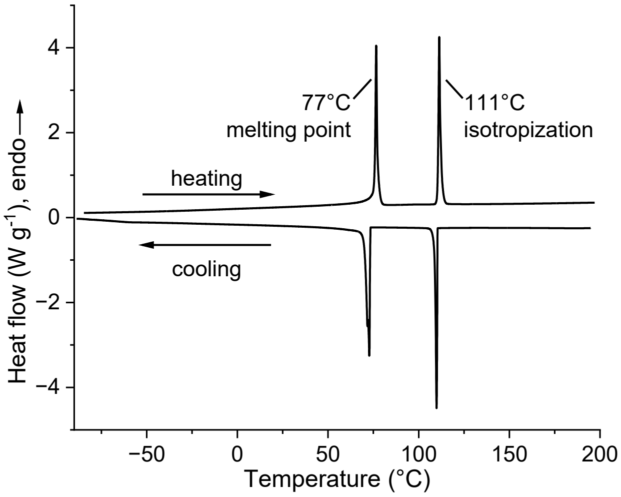

Supplementary Figure 7: *DSC measurement of the pure liquid crystalline monomer BPLC.*

Supplementary Note 3: Polymerization kinetics

Results of RT-NIR (real time near infrared photorheology) are displayed in **Supplementary Figure 8**. No measurements above 90 °C (measured on the measurement platform under the rheology stamp) could be performed as the heating elements of the rheometer would have to be heated beyond 120 °C, which could damage the UV light guide positioned below, in close proximity to the measurement platform. In addition to rheology, IR-spectra of the formulation were recorded during the curing process to monitor double bond conversion. However, due to crystallization a baseline shift occurred, which made it unfeasible to perform a baseline correction and calculate the double bond conversion. For each sample, three specimens were tested. The variations within the same temperature most likely arise from slightly differing amounts of formulation under the rheology stamp.

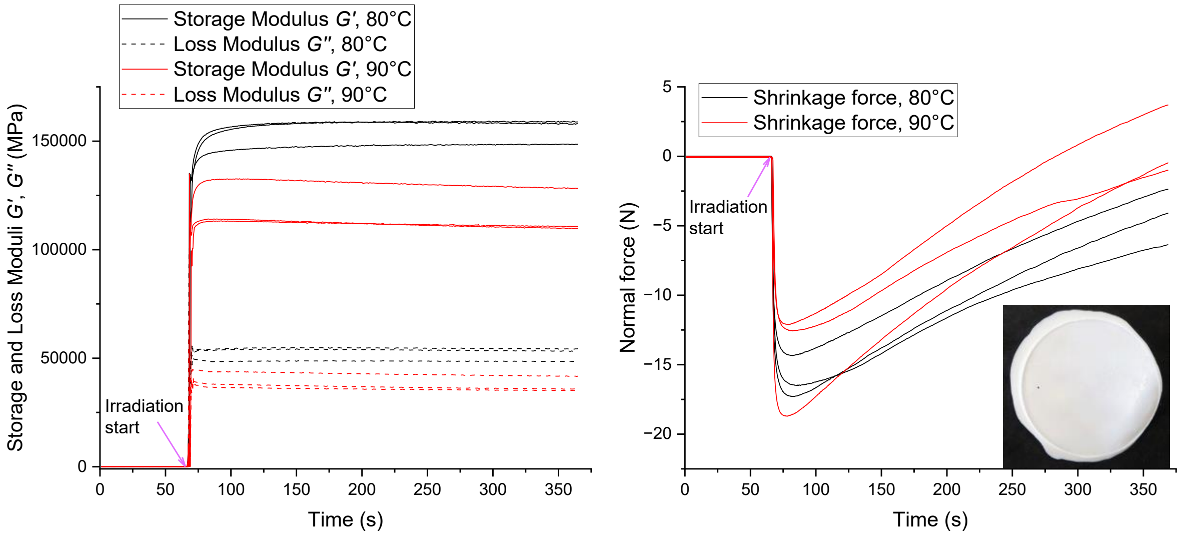

Supplementary Figure 8: *Real time photorheology measurement of formulation consisting of LCM5 and CHTT during the curing process. The 0.2 mm thick samples were irradiated using light from a 320-500 nm filtered mercury lamp with an intensity of 30 mW cm^{-2}. Irradiation starts at 65 seconds into the measurement. Measurements performed at 80 °C are depicted in black, measurements performed at 90 °C are depicted in red. A) Storage moduli depicted by solid lines, loss moduli depicted by dashed lines. B) Decrease in normal force during the course of the measurement (representative of shrinkage force/stress). The image of the inset shows the formed material after photorheology.*

Supplementary Note 4: Polymer crystallinity analysis

Results of differential scanning calorimetry analysis are listed in **Supplementary Table 3**. All data presented therein were recorded during the first heating cycle as minor shifts in melting points occurred during the second cycle. The crystallinity of polymers was compared quantitatively using the melting transition enthalpies of all DSC measurements where a baseline correction could be performed, which was the case for all samples except the ones bulk cured or printed at 110 °C (**Supplementary Figure 9**).

Supplementary Table 3: *Phase transition analysis of the monomer BPLC and various polymers cured from BPLC in conjunction with the thiol crosslinker CHTT. The phase transitions listed are the glass transition temperature T_g of polymers, melting point T_m, and the isotropization temperature T_i of the pure monomer. The combined enthalpy of melting ΔH_m and enthalpy of isotropization ΔH_i of the monomer BPLC were combined into the total phase transition enthalpy ΔH_{total}.*

Sample	T_g / °C	T_m / °C	T_i / °C	ΔH_m / J g^{-1}	ΔH_i / J g^{-1}	ΔH_{total} / J g^{-1}
BPLC (pure monomer)	-	77	111	49.1	41.3	90.4
Bulk crystalline (mild irradiation) 80°C	42	156	-	24.9	-	24.9
Bulk crystalline (strong irradiation) 80°C	19	142	-	0.84	-	0.84
Bulk amorphous 110°C	16	99	-	-[1]	-	-[1]
Printed crystalline 80°C	17	143	-	23.2	-	23.2
Printed amorphous 100°C	15	108	-	14.9	-	14.9
Printed amorphous 110°C	16	110	-	-[1]	-	-[1]
Printed 5-layer composite 80/100°C	16	145	-	10.1	-	10.1

[1]due to a broad and flat peak, no accurate baseline correction was possible to calculate the enthalpy of melting

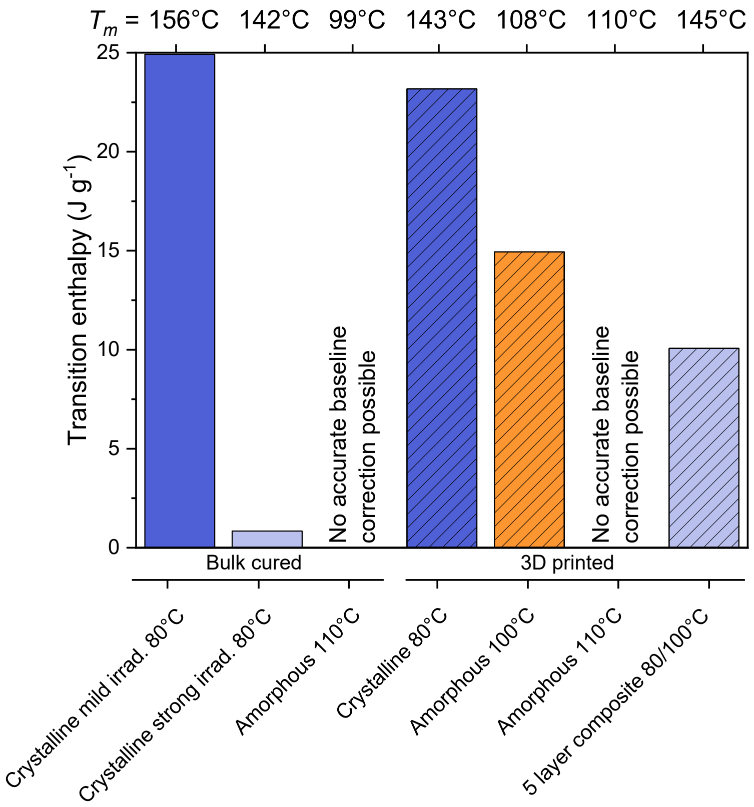

Supplementary Figure 9: *Phase transition enthalpies of polymers from the BPLC + CHTT formulation. Bulk cured samples are depicted as solid blocks, the sample cured at 80 °C with the mild irradiation procedure is depicted in dark blue, the sample cured with the strong irradiation procedure in light blue. Printed samples are depicted as textured blocks, the sample printed at 80 °C is depicted in dark blue, the sample printed at 100 °C in orange, the five layer composite in light blue.*

Supplementary Note 5: Dynamic mechanical analysis

The full dynamic mechanical analysis are displayed in **Supplementary Figures 10 and 11** for bulk cured and 3D printed samples, respectively. The samples cured at 80 °C display a distinct melting peak above 125 °C in the loss factor graph, while the samples cured at higher temperature exhibit a much more pronounced glass transition around room temperature with a less distinct melting peak at a lower temperature between 50 and 75 °C.

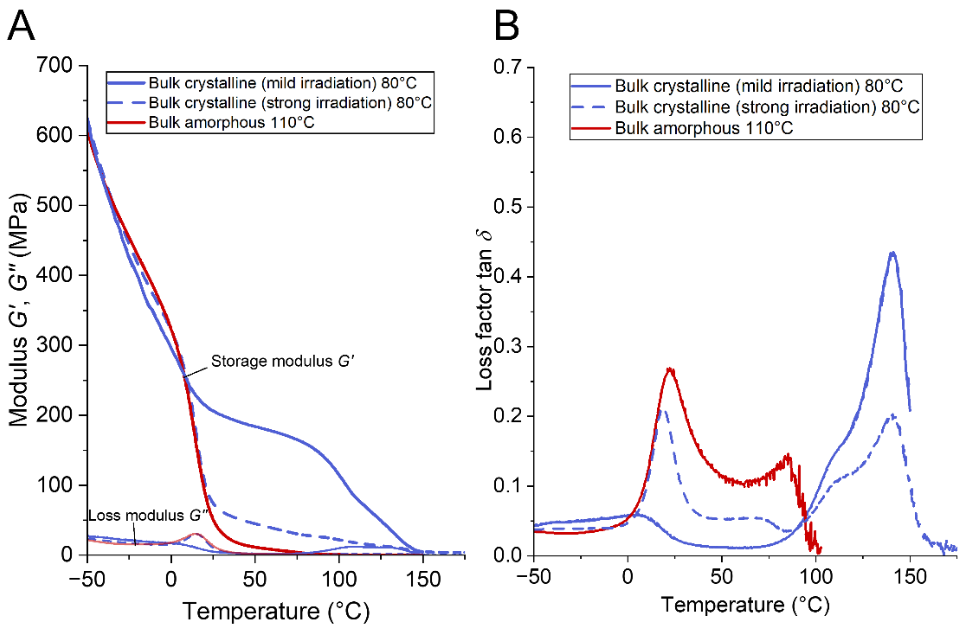

Supplementary Figure 10: *Full results of dynamic mechanical analysis (DMA) of bulk cured specimens. Samples were bulk cured at 80 °C with mild irradiation (blue solid line), 80 °C with strong irradiation (blue dashed line) and 110 °C (red line). A) Storage and loss modulus. B) Loss factor (tan δ), which is calculated by dividing the loss modulus by the storage modulus.*

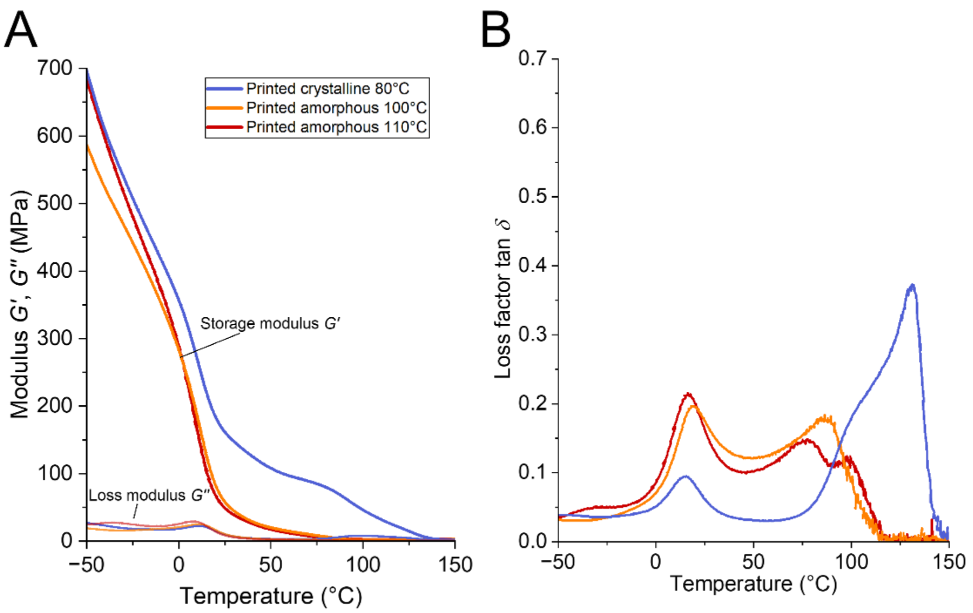

Supplementary Figure 11: *Full results of dynamic mechanical analysis (DMA) of 3D printed specimens. Samples were 3D printed at 80 °C (blue), 100 °C (orange) and 110 °C (red). A) Storage and loss modulus. B) Loss factor (tan δ), which is calculated by dividing the loss modulus by the storage modulus.*

Supplementary Note 6: Jacobs curves for the multi-temperature 3D printing process

The Jacob's curves (**Supplementary Figure 12**) suggest that the most important parameter influencing curing depth is light intensity rather than temperature. This reflects the difference in crystallization behaviour, which is favoured at low intensities as already found for bulk specimens. Therefore, penetration depth of light and therefore curing depth decreases over time, resulting in overall lower curing depth at lower intensity irradiation, even at similar exposure doses (i.e. shorter irradiation time for high intensity irradiation to match exposure of samples at lower intensity irradiation). The lack of temperature correlation with curing depth agrees with the fact that the polymerization kinetic are indistinguishable for all investigated temperatures from 80 °C on (see photorheology results in **Supplementary Figure 8**).

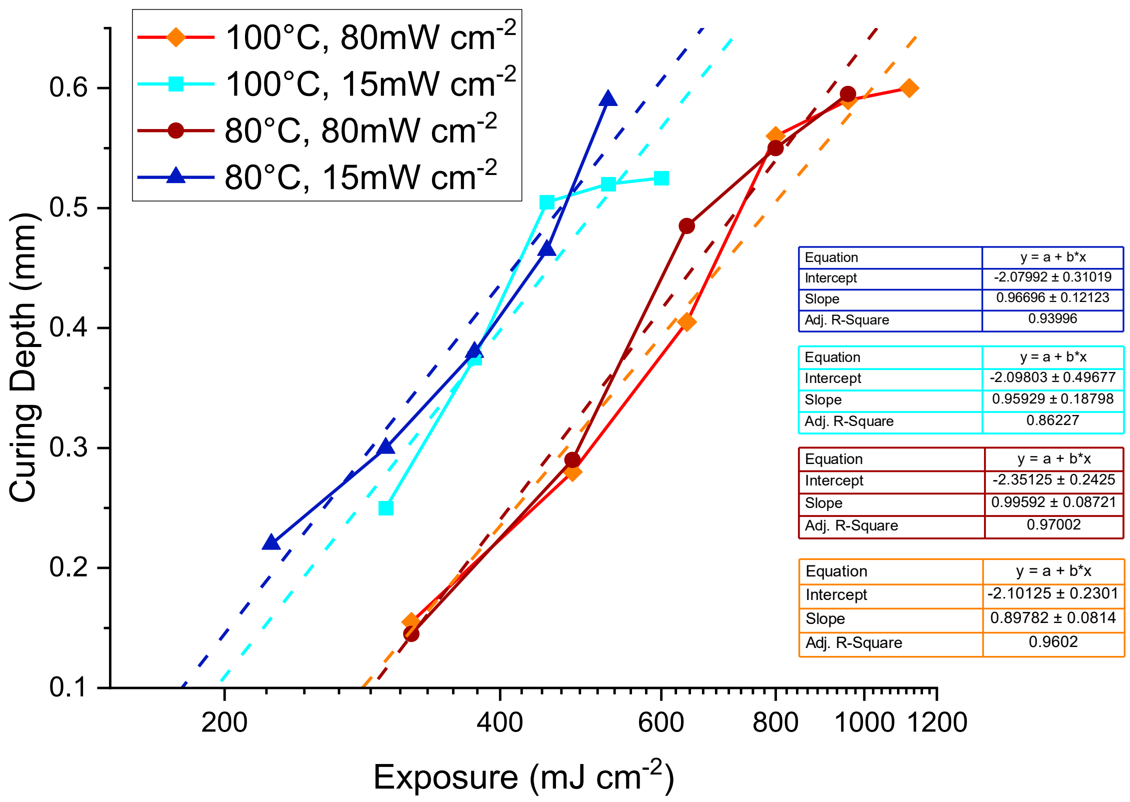

***Supplementary Figure 12:** Curing depths under different exposure conditions displayed as a Jacob's working curve. Depths were measured after removal of excess formulation using an electronic external measuring gauge (Kroeplin K110T). Samples irradiated at a temperature of 100 °C at an intensity of 80 mW cm^{-2} are depicted in orange with diamond shapes. Samples irradiated at a temperature of 100 °C at an intensity of 15 mW cm^{-2} are depicted in teal with square shapes. Samples irradiated at a temperature of 80 °C at an intensity of 80 mW cm^{-2} are depicted in red with circle shapes. Samples irradiated at a temperature of 80 °C at an intensity of 15 mW cm^{-2} are depicted in blue with triangle shapes. The fitting lines are depicted as dashed lines in the respective colours.*

Supplementary Note 7: Comparison samples with and without post-processing of printed specimens

Attenuated total reflection infrared (ATR-IR) spectroscopy measurements revealed high conversions directly after printing, especially for the transparent samples, which were printed at 100 and 110 °C (**Supplementary Figure 13**). The more crystalline sample printed at 80 °C showed a conversion around 90%. To investigate the necessity of post-curing quantitatively, the samples were irradiated in a

UVITRON IntelliRay UV chamber equipped with a 600W UVA Metal Halide Lamp (UV0834) at 100% intensity (approximately 180 mW cm^{-2}). The samples were post-cured for two minutes on each side. Measurements after post-curing indicate no statistically relevant changes in double bond conversions (calculated using [1]) for both the amorphous and crystalline samples. Therefore, it can be assumed that a close to final double bond conversion is achieved during the printing process, as relatively high irradiation intensities were used. The generally lower conversion for the crystalline samples could be caused by the lower curing temperature, which leads to a decreased reactivity. Thereafter, the crystallinity inhibits the mobility of the reactive chains in the forming polymer network, causing the conversion to not increase further during post-curing.

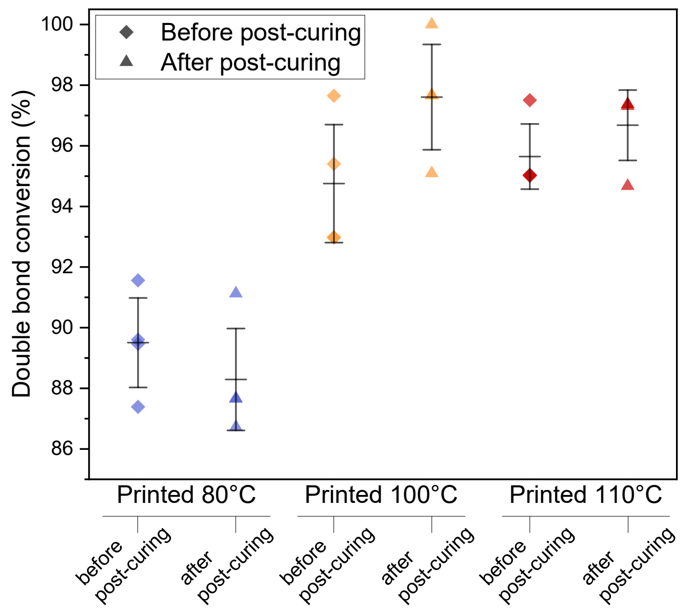

Supplementary Figure 13: *Double bond conversion via ATR-IR-spectroscopy of printed specimens before and after post-curing. Samples were 3D printed at 80 °C (blue), 100 °C (orange) and 110 °C (red). Non-post-cured samples are displayed as diamonds, post-cured samples are displayed as triangles. The error bars display the mean ± SD, n=4.*

The effect of post-curing with UV light on printed parts was tested by subjecting the specimens, either amorphous, crystalline or in multi-material form, to post-curing. As demonstrated in **Supplementary Figure 14**, we were unable to find any change in optical properties. This was further confirmed quantitatively by near-identical DSC curves before and after post-curing (**Supplementary Figure 15**).

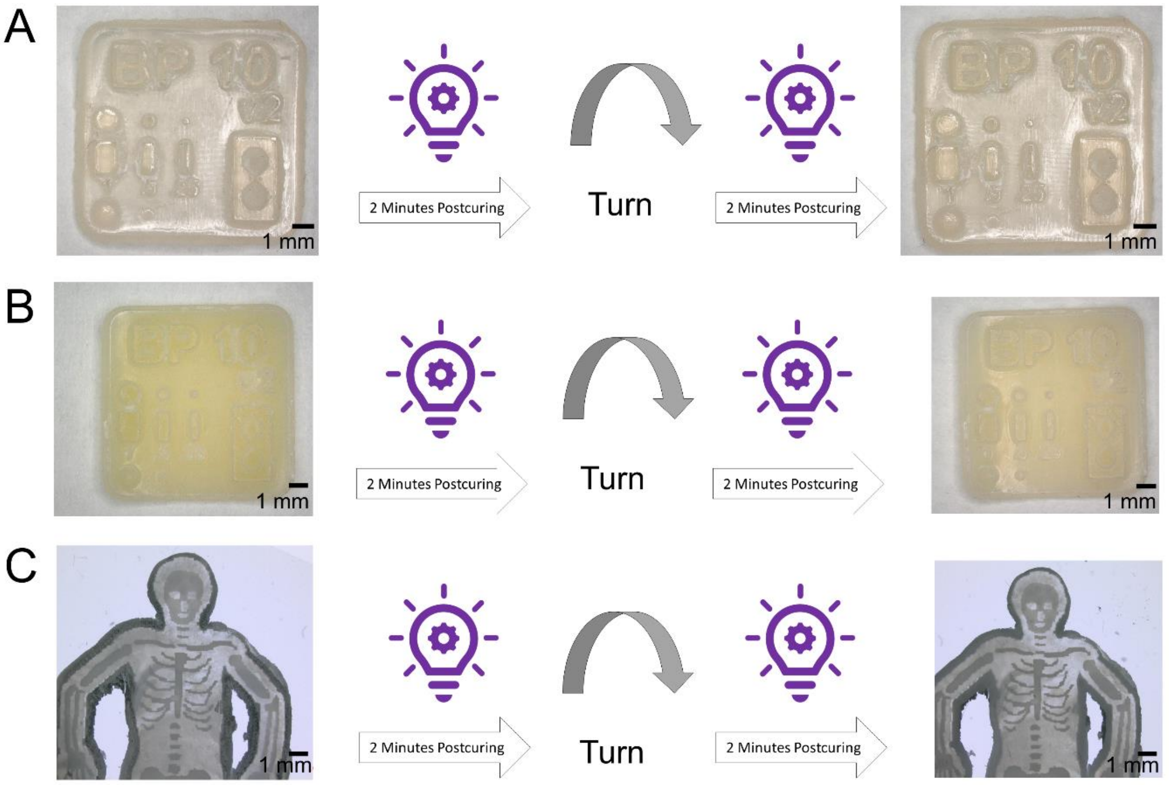

Supplementary Figure 14: *Effect of post-curing different printed parts for 2 minutes on each side.*

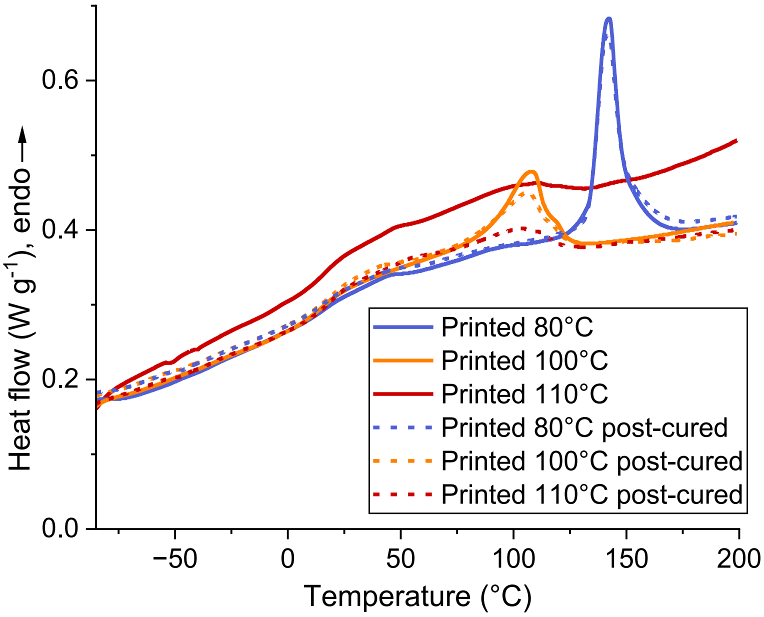

Supplementary Figure 15: *DSC of 3D printed polymer samples before and after post-curing. Samples were 3D printed at 80 °C (blue), 100 °C (orange) and 110 °C (red). Post-cured samples are displayed as dashed lines.*

Supplementary Note 8: Gel fraction

The gel fraction of the samples was determined by weighing them after polymerization, then after swelling them in toluene for 24 h and after drying them to a constant weight (**Supplementary Figure 16**). After removal from the solvent, the sample printed at 80 °C had swollen only slightly, while both amorphous samples had swollen to nearly double their original mass. After drying, a gel fraction over 97% was achieved for all samples.

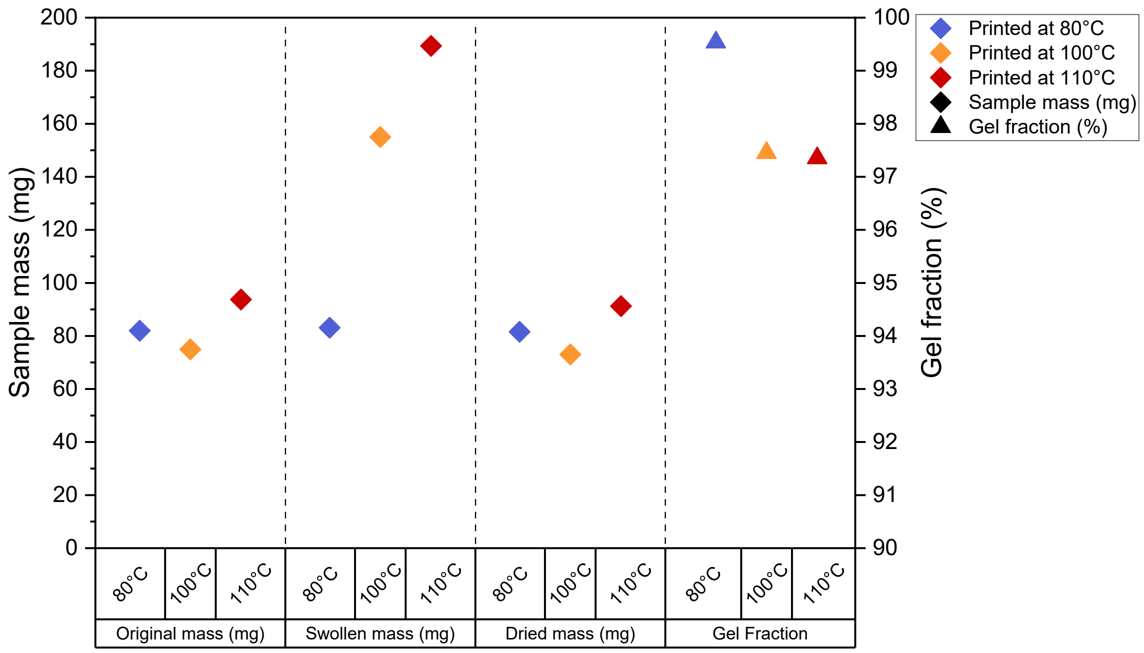

Supplementary Figure 16: *Determination of gel fraction from original sample mass, sample mass when swollen with toluene, and dried mass after swelling. Samples were 3D printed at 80 °C (blue), 100 °C (orange) and 110 °C (red). The gel fraction is determined from the ratio of dried mass and original mass.*

Supplementary Note 9: Polymer density and shrinkage analysis

The density measurement via Archimedes method revealed very similar densities for all three used printing temperatures, from which polymerization shrinkages were calculated, which ranged from approximately 2.5 to 3% (**Supplementary Figure 17**).

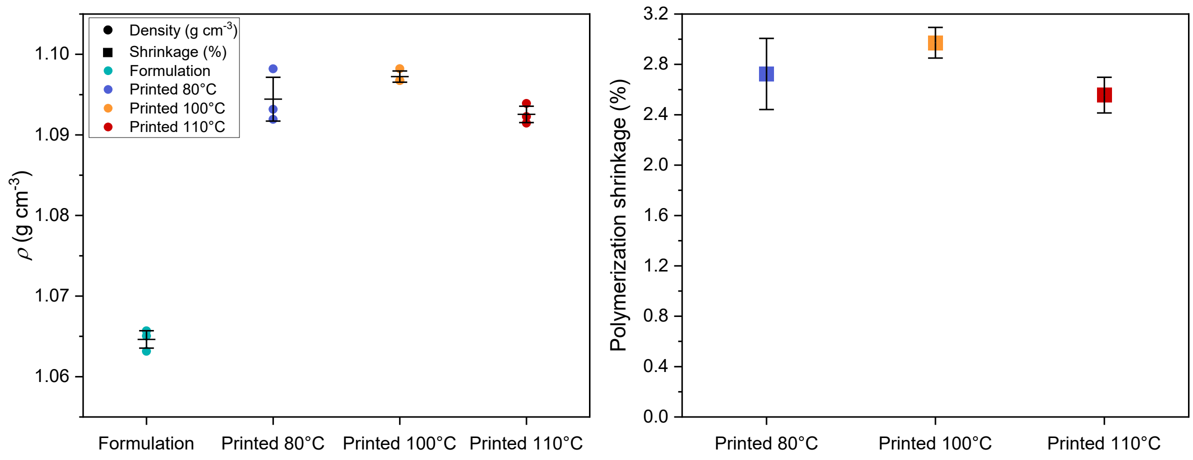

Supplementary Figure 17: *A) Densities ρ of unpolymerized formulation (dark teal) and 3D printed polymer samples measured via the Archimedes method depicted as circles. Samples were 3D printed at 80 °C (blue), 100 °C (orange) and 110 °C (red). The error bars display the mean ± SD, n=3. B) Polymerization shrinkages calculated from the ratio of formulation and polymer density depicted as squares. For the calculation of polymerization shrinkage standard deviations, Gauss's law of error propagation was utilized.*

Supplementary Note 10: Temperature gradient print and irradiation intensity test

Cryo-microtome cuts of the temperature gradient 3D print were performed using a glass knife at a thickness of 10 µm at -80 °C. **Supplementary Figure 18** shows a full microtome slice of the printed sample, which was subjected to a temperature gradient and two different irradiation intensities and times (80 mW cm^{-2}, 4.7 s and 15.75 mW cm^{-2}, 24 s, same total light dose). The approximate total printing time for each layer in this test was 34 s, which includes irradiation and the raising and lowering of the printing platform. Herein, the polarizing filter is in a slightly different rotation than in the picture displayed in the main paper, which resulted in the bright phenomena caused by the microtome cut shifting to different locations. After every 4 layers, the temperature was increased by 2 °C. A "bleeding" phenomenon is observed between the high- and low-irradiation intensity sections. The most likely cause of this is crystallite nucleation, which propagates throughout the layer from the more crystalline, low irradiation intensity side. Thus, the nearly fully amorphous layer between 81 and 82 °C on the higher irradiation intensity site, which is followed by a number of slightly crystalline layers could be caused by a randomly occurring lack of nucleation sites. Alternatively, high irradiation intensity could cause significantly higher exothermic behaviour during 3D printing, thus exceeding the liquid crystalline temperature range and hence inhibiting the preordering of liquid crystalline building blocks. This is of course more likely to occur close to the upper liquid crystalline temperature regime threshold, and at high intensity irradiation.

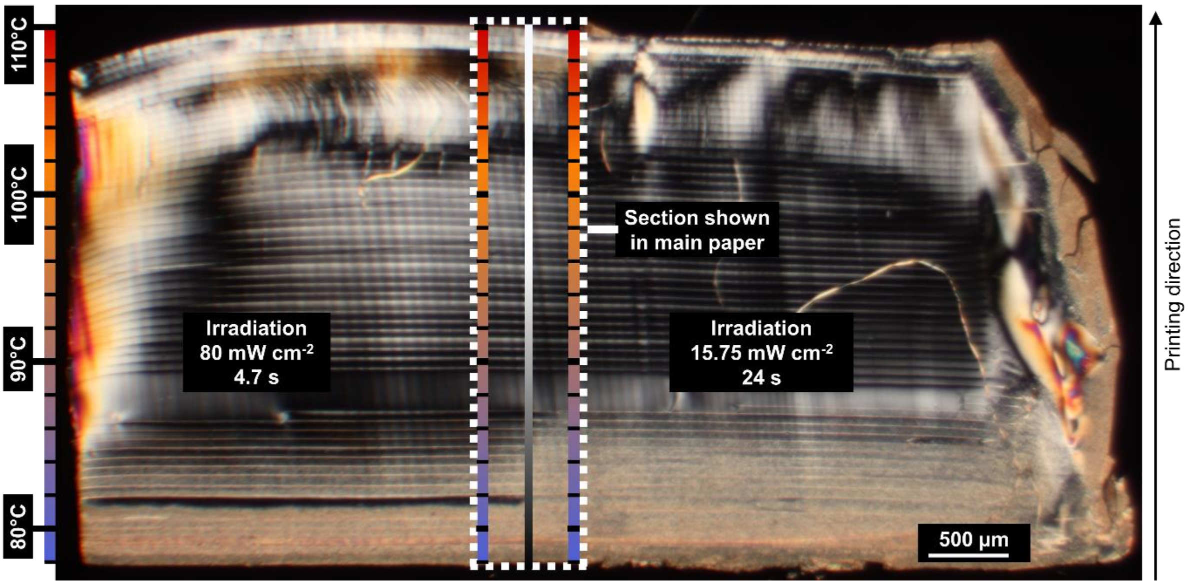

Supplementary Figure 18: *Microtome slice of the gradient print, which was irradiated at two different irradiation intensities within each layer with progressively increasing temperatures. Every 4 layers, the temperature was increased by 2 °C. The black to white gradient line separates the sections irradiated at different intensities.*

Supplementary Note 11: Transparency evaluation

A polymer sample printed at 85 °C, in which a variation of crystallinity was purely caused by a change in irradiation parameters on different sections (greyscale printing), was investigated using transmission IR and visible light spectroscopy (**Supplementary Figure 19**). The measurement was repeated after a post-curing step, which led to a minor difference in the low-wavelength range, which is likely caused by the cleavage of products of the photoinitiator. Across the remainder of the wavelength range, no change could be observed and the transmittance and the attenuation coefficient remained unchanged after post-curing.

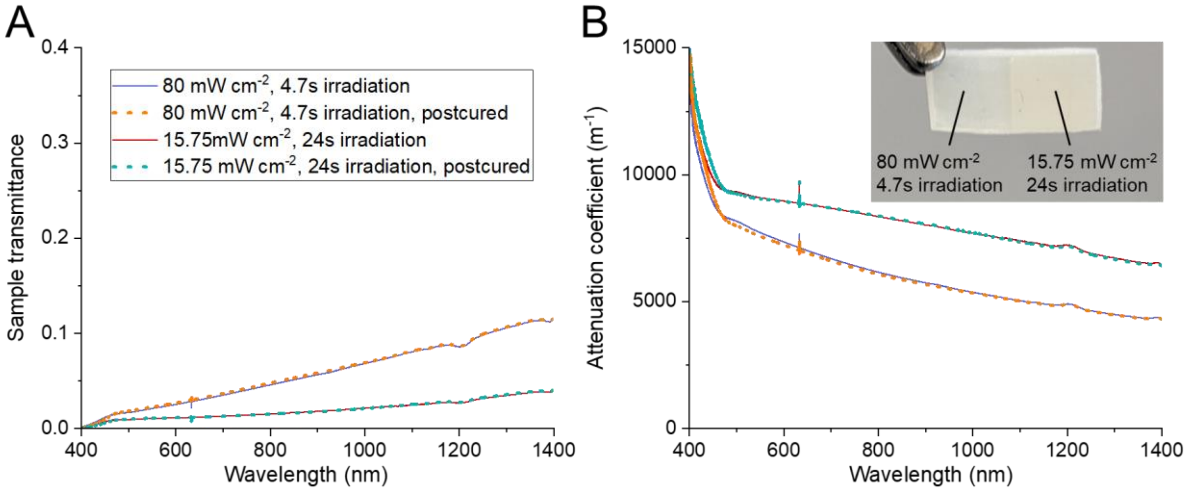

Supplementary Figure 19: *Transmittance and attenuation coefficient of two 5x5 mm sides of a polymer sample printed at 85 °C with two different irradiation intensities and no temperature changes. The measurements were performed before and after a post-curing step to demonstrate that the optical sample properties were unaffected by post-curing. The sample irradiated at 80 mW cm^{-2} for 4.7 s is depicted in solid blue before post-curing and dotted orange after post-curing. The sample irradiated at 15.75 mW cm^{-2} for 24 s is depicted in solid red before post-curing and dotted teal after post-curing.*

Additionally, two separate printed samples, one crystalline specimen printed at 80 °C and one amorphous sample printed at 100 °C, were investigated using transmission IR and visible light spectroscopy to quantify the difference in light absorption across the visible and near-infrared range (**Supplementary Figure 20**).

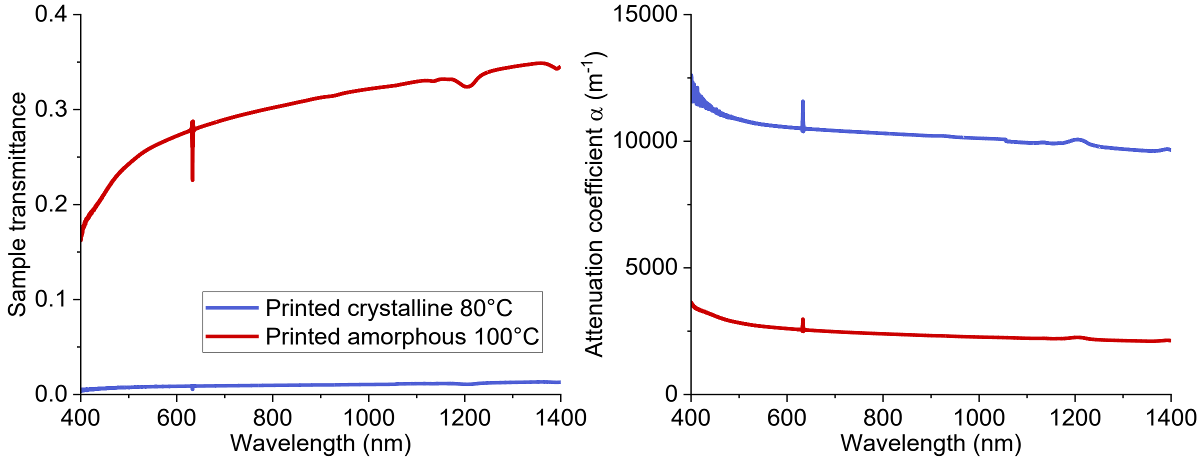

Supplementary Figure 20: *Transmittance and attenuation coefficient of 3D printed polymer samples measuring 8x8x0.5 mm³. An artifact of the internal helium-neon reference laser in the FTIR instrument is observed at 633 nm. The sample printed at 80 °C is depicted in blue, the sample printed at 100 °C is depicted in red.*

Supplementary Note 12: 3D printing of multi-material parts

Supplementary Figure 21 depicts the heating and cooling cycle for one multi-material print layer. The period of time needed to heat and cool the printer's build plate to the temperatures required for multi-temperature printing is significantly longer than the time needed to heat and cool the material vat. With the implementation of more effective heating elements and an efficient active cooling setup, a printing time of less than two minutes per layer could be achieved.

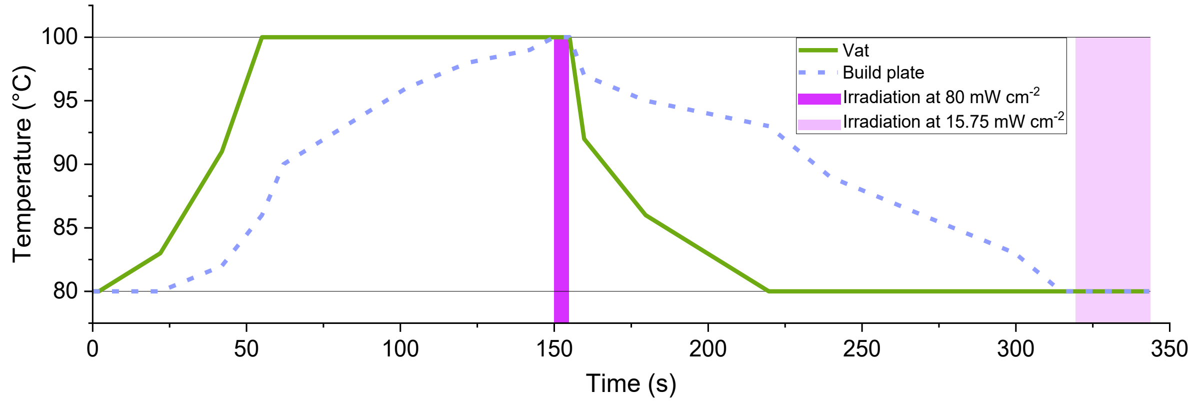

Supplementary Figure 21: *Graphical representation of the temperatures in the vat and on the printing platform during printing of a multi-material layer. The required times for heating and cooling were measured with the same setup that was used to print the parts. Room temperature was 21 °C during this measurement. The vat temperature is depicted in solid green, the build plate temperature as dashed blue. The higher-intensity irradiation period is depicted as a dark purple area, the lower-intensity irradiation period as a light purple area.*

Supplementary Note 13: Dimensional accuracy

We conducted several measurements to find the dimensional accuracy of differentiation between amorphous and crystalline sections achievable in in multi-material layers. In **Supplementary Figure 22**, measurements of a single element of the QR Code sample revealed a deviation of 16 µm in X-direction and 17 µm in Y-direction. Additionally, the "mouth" of the skeleton model exhibited deviations of 4 and 7 µm in the X-direction and 22 µm in the Y-direction. The difference between X and Y may be explained since the model was thin and twisted during removal from the build plate. Finally, we provide a graphical overlay to compare the digital model with the printed model. A deviation can again be observed in the skeleton model, which can be explained by the warping of the thin model during removal from the build plate. Throughout the measurements, the deviations from the model overlay are mostly caused by warping of the printed specimens. Even so, no deviations were found, which were larger than half a pixel-pitch width (each pixel represents a square 50x50 µm² area).

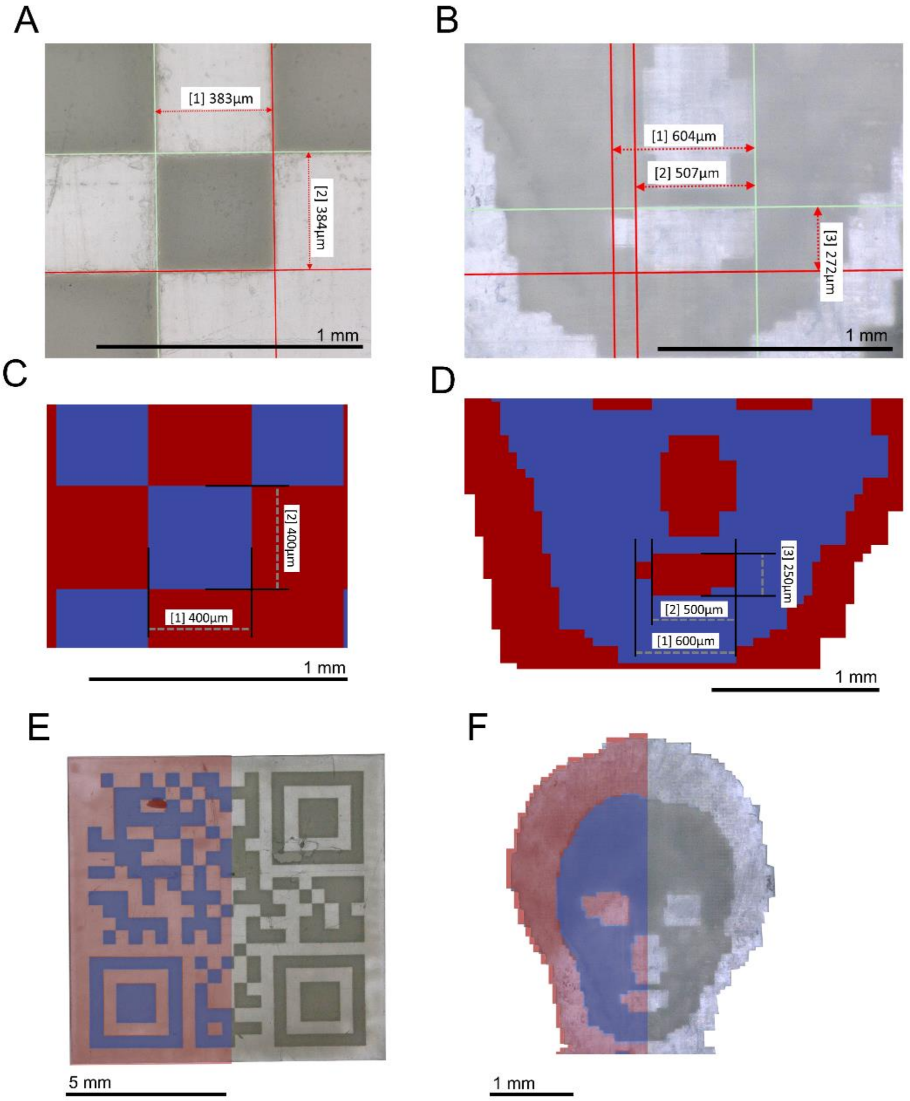

Supplementary Figure 22: *Dimensional accuracy measurements of multi-material printed samples. A) Single element of the QR code sample with a size of 8x8 pixels or 400x400 µm as designed. The printed part shows a deviation of 16 µm in X direction (measurement [1] displayed in the image) and 17 µm in Y direction (measurement [2]). B) Measurements on the "mouth" of the skeleton model show a deviation of 4 µm(measurement [1]) and 7 µm (measurement [2]) in X direction and 22 µm in Y direction (measurement [3]). For C-F, the parts printed at 80 °C are depicted in blue, the parts printed at 100 °C in red. C) Digital model of the shown QR code section. D) Digital model of the shown section of the skeleton model. E) Image of the complete QR code sample under transmitted light microscopy. The left half of the image is overlayed with the digital design to show the accuracy that can be achieved over a larger object. F) Detail image of the head of the skeleton model under transmitted light microscopy. The left half is overlayed with the digital design that was used for printing the model.*

Supplementary Note 14: Shape Memory test results

Supplementary Table 4 and **Supplementary Figure 23** contain the results of the conventional shape memory measurements, in which the sample is elongated to 50% strain in the first cycle and to the same absolute length in subsequent cycles. **Supplementary Table 5** and **Supplementary Figures 24-26** contain the results of the modified method, in which the sample is elongated to 50% strain in the first cycle and elongated by the same strain in subsequent cycles.

Supplementary Table 4: *Results of conventional shape memory tests. Herein, the sample was elongated to 50% elongation relative to the base length of the parallel sample section each time (starting parallel section length 12mm, parallel section length at 50% elongation 18mm). Herein, for the first cycle, equation [5] was used to calculate the shape recovery ratio, and for the other cycles, which exhibited a shape fixity ratio above 100%, equation [4] was used to calculate the shape recovery ratio.*

Conventional measurement technique

Cycle number	Shape Fixity Ratio (%)	Shape Recovery Ratio (%)
1	99.8	79.5
2	100.1	96.3
3	100.2	97.8
4	100.2	98.3
5	100.3	98.6
6	100.4	98.8
7	100.4	98.9
8	100.4	99.1

Supplementary Table 5: *Results of shape memory tests from testing the same sample three times. Herein, a modification was applied, for which the elongation was set to 0% after each cycle, causing an elongation of the sample over time.*

Modified measurement technique

	First measurement		Second measurement		Third measurement	
Cycle number	Shape Fixity Ratio (%)	Shape Recovery Ratio (%)	Shape Fixity Ratio (%)	Shape Recovery Ratio (%)	Shape Fixity Ratio (%)	Shape Recovery Ratio (%)
1	100.3	99.8	100.9	100.9	101.0	101.7
2	100.5	98.2	100.7	99.1	101.0	99.7
3	100.6	99.4	100.9	100.1	101.0	99.9
4	100.6	99.2	100.9	99.3	101.1	99.9
5	100.6	98.2	100.9	99.9	101.0	100.0
6	100.6	99.8	101.0	100.0	101.0	99.9
7	100.8	99.2	100.9	99.6	101.0	99.7
8	100.8	99.8	101.0	99.7	101.0	99.7
9	100.8	99.5	100.9	100.0	101.0	100.1
10	100.8	99.8	101.0	99.9	101.1	99.9

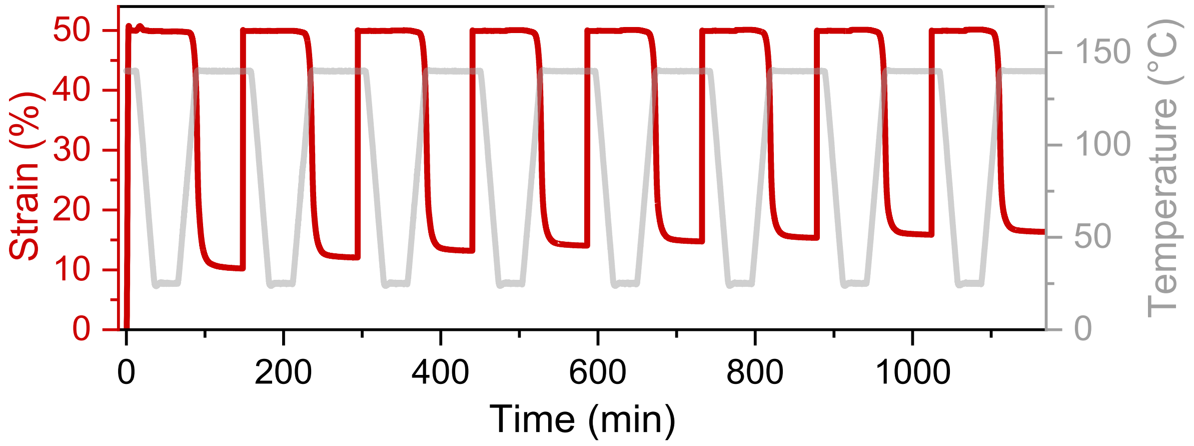

Supplementary Figure 23: *Eight-cycle shape memory evaluation using the conventional method. The strain of the sample during the measurement is depicted in red, the temperature in light grey.*

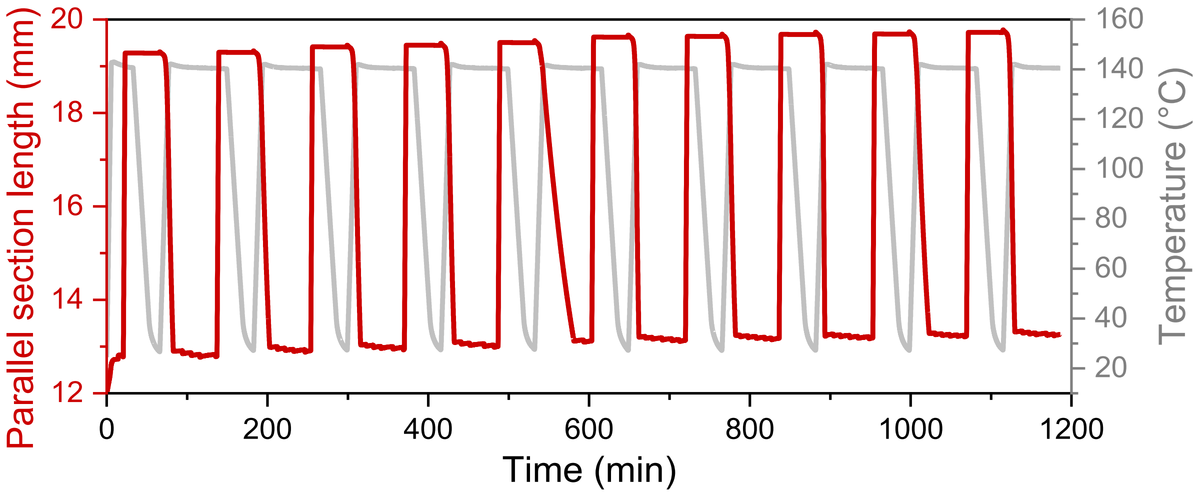

Supplementary Figure 24: *First modified technique ten-cycle shape memory test. The length of the parallel section of the tensile test specimen is depicted in red, the temperature in light grey.*

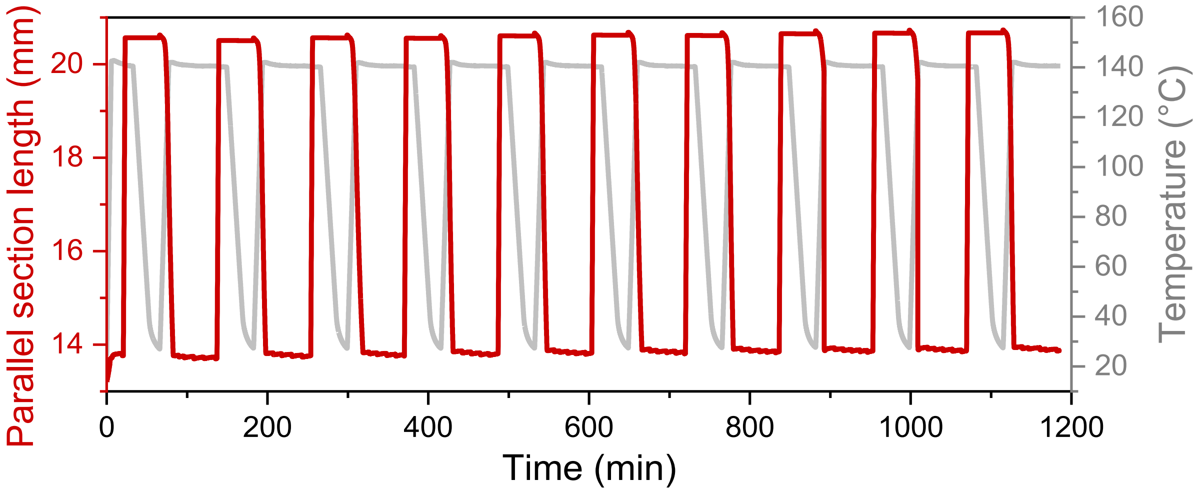

Supplementary Figure 25: *Second modified technique ten-cycle shape memory test. The length of the parallel section of the tensile test specimen is depicted in red, the temperature in light grey.*

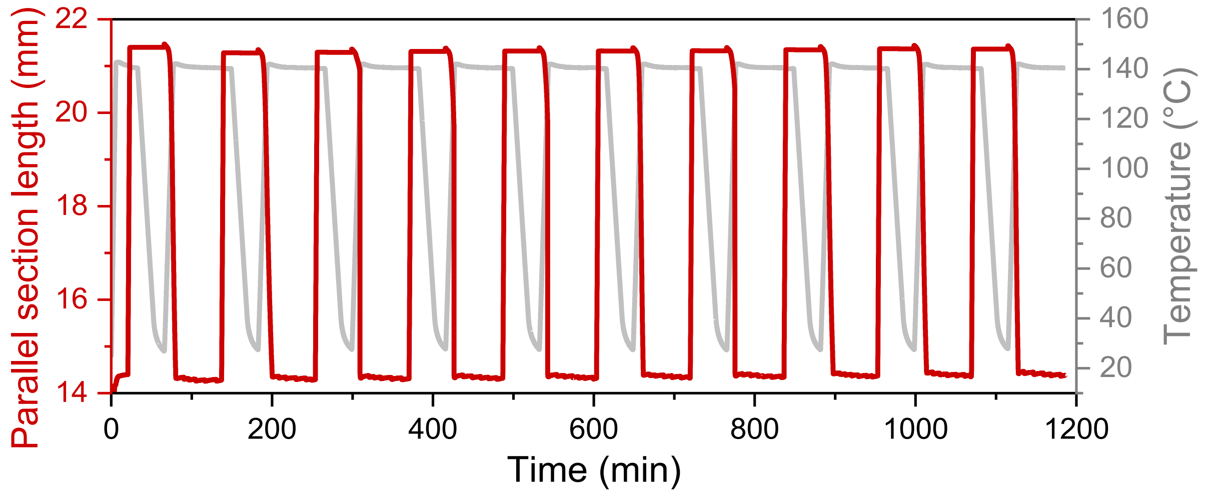

Supplementary Figure 26: *Third modified technique ten-cycle shape memory test. The length of the parallel section of the tensile test specimen is depicted in red, the temperature in light grey.*
