# Supplementary material for: Semi-crystalline and amorphous materials via multi-temperature 3D printing from one formulation
Source: Nat Commun. 2025 Oct 15;16:8961. doi: 10.1038/s41467-025-64092-9 (PMC12528444; doi:10.1038/s41467-025-64092-9)
Supplement: Supplementary file 2 — Description of Additional Supplementary Files [file 41467_2025_64092_MOESM2_ESM.pdf]

Description of Additional Supplementary Files

Supplementary Movie 1: Reveals process of the multi-temperature printed QR code, which is obscured by a crystalline cover layer. Uniform heating from the top, achieved here by placing a glass slide on top of the printed specimen and heating it evenly using a heat gun, melts the crystalline sections of the top layer, revealing the crystalline/amorphous multimaterial QR code structure, which can be scanned to reveal the phrase “3D-Encoding”.
